# Comparison of the Mitochondrial Genome Sequences of Six *Annulohypoxylon stygium* Isolates Suggests Short Fragment Insertions as a Potential Factor Leading to Larger Genomic Size

**DOI:** 10.3389/fmicb.2018.02079

**Published:** 2018-09-10

**Authors:** Youjin Deng, Tom Hsiang, Shuxian Li, Longji Lin, Qingfu Wang, Qinghe Chen, Baogui Xie, Ray Ming

**Affiliations:** ^1^Center for Genomics and Biotechnology, Haixia Institute of Science and Technology, College of Life Sciences, Fujian Agriculture and Forestry University, Fuzhou, China; ^2^Department of Plant Biology, University of Illinois at Urbana-Champaign, Urbana, IL, United States; ^3^Environmental Sciences, University of Guelph, Guelph, ON, Canada; ^4^USDA-Agricultural Research Service, Crop Genetics Research Unit, Stoneville, MS, United States

**Keywords:** intron insertion site, Twintron, ORF-less intron, small inverted repeat, mitochondrial genome

## Abstract

Mitochondrial DNA (mtDNA) is a core non-nuclear genetic material found in all eukaryotic organisms, the size of which varies extensively in the eumycota, even within species. In this study, mitochondrial genomes of six isolates of *Annulohypoxylon stygium* (Lév.) were assembled from raw reads from PacBio and Illumina sequencing. The diversity of genomic structures, conserved genes, intergenic regions and introns were analyzed and compared. Genome sizes ranged from 132 to 147 kb and contained the same sets of conserved protein-coding, tRNA and rRNA genes and shared the same gene arrangements and orientation. In addition, most intergenic regions were homogeneous and had similar sizes except for the region between cytochrome b (cob) and cytochrome c oxidase I (cox1) genes which ranged from 2,998 to 8,039 bp among the six isolates. Sixty-five intron insertion sites and 99 different introns were detected in these genomes. Each genome contained 45 or more introns, which varied in distribution and content. Introns from homologous insertion sites also showed high diversity in size, type and content. Comparison of introns at the same loci showed some complex introns, such as twintrons and ORF-less introns. There were 44 short fragment insertions detected within introns, intergenic regions, or as introns, some of them located at conserved domain regions of homing endonuclease genes. Insertions of short fragments such as small inverted repeats might affect or hinder the movement of introns, and these allowed for intron accumulation in the mitochondrial genomes analyzed, and enlarged their size. This study showed that the evolution of fungal mitochondrial introns is complex, and the results suggest short fragment insertions as a potential factor leading to larger mitochondrial genomes in *A. stygium*.

## Introduction

The mitochondrial organelle is found in most eukaryotic organisms (Karnkowska et al., 2016). Mitochondria are thought to have had a monophyletic origin from a vestigial endosymbiotic proteobacterial ancestor, but the size and the gene content of mitochondria are much less than those of free-living proteobacteria (Adams and Palmer, 2003). Some mitochondrial genes have been transferred to the host nucleus, while other genes have been lost (compared to extant proteobacteria), likely because there are genes with similar function in the nuclei of eukaryotes (Adams and Palmer, 2003). Common characteristics of mitochondrial DNA (mtDNA) are the high A+T content, lack of methylation, conservation of the gene function, and high copy number (Campbell et al., 1999).

With the rapid development of high-throughput sequencing technologies, increasingly more fungal mitochondrial genomes have been sequenced and characterized. The reported genomes usually contain a similar set of conserved genes, including 14 protein-coding genes (*nad1, nad2*, nad3, *nad4, nad4L, nad5, nad6, atp6, atp8, atp9, cox1, cox2, cox3*, and *cob*), a set of tRNAs, and small and large ribosomal RNA (*rns* and *rnl*). However, the size of fungal mtDNAs varies greatly. The smallest mitochondrial genomes, such as those of *Harpochytrium* sp. and *Candida orthopsilosis*, are around 20 kb in size with few or no introns and few or no non-conserved genes. Several large mitochondrial genomes in fungi (more than 150 kb) have been released in the organelle genome database in NCBI recently, including those of *Rhizoctonia solani* AG-1 and AG3 (Losada et al., 2014), *Sclerotinia borealis* F-4128 (Mardanov et al., 2014), *Pyronema omphalodes* CBS 100304 (Nowrousian, 2016), *Chrysoporthe austroafricana* (Kanzi et al., 2016), *Ustilago bromivora* UB2112, *Phellinus noxius* FFPRI411160, *Ophiocordyceps sinensis* (Kang et al., 2017), and *Phlebia radiata* 79 (Salavirta et al., 2014). One common features among large fungal mtDNAs is that they contain many introns. Comparison of other mtDNAs also reveals that intron number and length is positively correlated to mtDNA size, and is the primary source of size variation (Deng et al., 2016b). At present, the mechanisms of why and how larger mtDNAs evolved and more introns were retained remains unknown.

Mitochondrial introns usually consist of a catalytically active intron RNA and a small intron-encoded protein (IEP; Michel and Ferat, 1995). The RNA functions as a ribozyme to catalyze its own splicing, yielding spliced exons and an excised intron. Based on RNA secondary structure, introns in fungal mtDNAs are classified into two major groups: I and II (Saldanha et al., 1993). Group I introns are subdivided into seven specific types: IA, IA3, IB, IC1, IC2, ID, and group I derived (I^*^) (Lang et al., 2007). Group I introns are widely distributed, probably through horizontal transfer, and the driving force is presumed to be supplied by homing endonuclease (HE) genes, which they carry or are from the host (Gonzalez et al., 1998). The HE genes encode enzymes to promote intron mobility via introducing doubled stranded breaks at specific target sites. Based on conserved amino acid motifs, two families of HE genes are usually encoded by the open reading frame (ORF) of Group I introns, namely the LAGLIDADG and GIY-YIG proteins. The IEP of the group II intron is a multifunctional reverse transcriptase, which stabilizes the catalytically active RNA structure when splicing, and reverse transcribes its hosts intron into new DNA (Lambowitz and Zimmerly, 2011).

Twintron refers to a intron complex with an existing intron containing an insertion of a mobile intron (Bullerwell et al., 2003). This term was first proposed by Copertino and Hallick (Copertino and Hallick, 1991) based on chloroplast *psbF* gene research in *Euglena gracilis*. Since then, several categories of twintrons have been identified. According to the group types of internal and external introns, a twintron can be group I internal to group I (Einvik et al., 1998), group II internal to group II (Copertino and Hallick, 1991), group I internal to group II (Hafez et al., 2013), and group II internal to group I. A twintron can also be a complex of multiple introns, with excisions yielding three or more individual introns (Drager and Hallick, 1993). Although major twintrons have been reported in the chloroplast genome of *Euglena*, the genetic elements have also been uncovered recently in fungal mitochondrial genomes. Two twintrons have been detected in the positions of mS917 and mS1247 in the *rns* gene of the *Cryphonectria parasitica* mitochondrial genome. The mS917 twintron is an ORF-less group ID intron invaded by another ID intron encoding LAGLIDADG in the ORF. The mS1247 twintron was the first recorded mixed twintron among fungal mitochondrial genomes, and could be excised into a group II internal intron and a group I external intron (Hafez et al., 2013). *Cox3*-i2 in mtDNA of *Hypomyces aurantius* H.a 0001, is a new twintron complex (Deng et al., 2016b). Instead of one invaded by the other, the two group IA introns within this twintron complex are arranged side by side (Deng et al., 2016b).

The genus *Annulohypoxylon*, previously referred to as *Hypoxylon* sect. Annulata (Suwannasai et al., 2013), belongs to Xylariaceae, Ascomycota. Among its 45 currently accepted species, many have been isolated from plants as endophytes or as pathogens (Stone et al., 2004). *Annulohypoxylon*, as well as other genera in Xylariaceae has attracted natural products researchers in recent decades for the high diversity of secondary metabolites, which are highly specific for certain taxa or taxonomic groups as chemotaxonomic markers (Whalley and Edwards, 1995). Aside from identification of natural products for taxonomic and medicinal objectives (Maciel et al., 2017), *A. stygium* has been reported to be a potential candidate for glycol hydrolase production due to its high pectinase, xylanase and β-glucosidase activities under specific conditions (Robl et al., 2015). *A. stygium* is also identified as associated with *Tremella fuciformis*, providing nutrition for growth and development of the commercially valued fruit bodies (Deng et al., 2016a).

In this study, the complete mitochondrial genome sequencing of six isolates of *A. stygium* was conducted. Mitochondrial reads were isolated, and assembled into complete circular molecules. Mitochondrial genome comparisons were performed to analyze the diversity of genomic structures, conserved genes, intergenic regions and introns. The purpose of the study was to elucidate the evolution of complex introns, and elucidate possible forces driving mitochondrial genome enlargements in *A. stygium*.

## Materials and methods

### Isolates and genome sequencing

Six isolates of *A. stygium* were used in this study. Isolate As31 was obtained from the Edible Fungal Germplasm Resources Management Center of Fujian province, Fuzhou, China. It is a companion fungus of a major *T. fuciformis* commercial cultivar Tr21. The other five isolates, As03, As15, As23, As24, and As28 were isolated from rotting wood substrates associated with naturally growing *T. fuciformis* fruit bodies in different areas of Fujian, China. Following Hsieh et al. (2005), concatenation of beta-tubulin and actin gene sequences was used for species designations of the isolates. Corresponding sequences of *Annulohypoxylon urceolatum* (AY951670 and AY951782), *A. stygium* 90081906 (AY951667 and AY951775), *A. stygium* 90041409 (AY951666 and AY951776), *A. stygium* var. annulatum 3 (AY951669 and AY951777) and *A. stygium* var. annulatum 91042205 (AY951668 and AY951778) were employed as controls. Sequences were aligned using Clustal W with gap opening penalty of 5 and gap extension penalty of 2. The distances were computed using the Neighbor-joining method. All positions containing gaps and missing data were eliminated from phylogenetic analysis. Evolutionary analyses were conducted in Mega 7 (Kumar et al., 2016). After growth in potato dextrose broth at 28°C and 180 rpm for 48 h, the hyphae were collected by filtration through Whatman #1 filter paper, and washed three times with sterilized distilled water. Total genomic DNA was isolated using the CTAB method (Manicom et al., 1987).

PacBio Sequencing was used to obtain a complete mitochondrial genome of As31 as well as the nuclear genome. Three SMRT cells were used in the PacBio RS II system, to obtain 3.5 GB raw data with a mean read length of 4.8 kb. In addition, an Illumina sequencing strategy was used to sequence all six isolates. Paired-end sequencing libraries with an insert size of ~500 bp were constructed, and then sequenced to 3–4 Gb raw data per isolate with 250 bp paired-end reads using the Illumina Hiseq 2500 system. All sequencing was done at the Genome and Biotechnology Research Center, Fujian Agriculture and Forestry University.

### Genome assembly

The PacBio reads of As31 were assembled into contigs using the software Canu 1.2 (Koren et al., 2017). Mitochondrial related contigs were identified using local BLASTX against a database of known mitochondrial proteins from closely related species. The contigs were assembled into a finished circle molecule by overlap manually. Some small indels or polymorphisms were corrected by mapping short reads from Illumina sequencing onto the PacBio genome. Illumina reads of the five other isolates were assembled into draft genome sequences using the software SPAdes-3.8.1 (Bankevich et al., 2012). Mitochondrial related contigs were isolated via doing BLASTN of whole genomic contigs against mtDNA of As31, and then assembled into circular molecules using paired-end read relationships. Remaining gaps were resolved by conventional PCR with Sanger sequencing.

### Genome annotation

Annotation of mitochondrial genomes was performed using the automated annotation program MFannot (http://megasun.bch.umontreal.ca/cgi-bin/mfannot/mfannotInterface.pl). Borders between exons and introns were confirmed manually using BLASTP against NCBI protein databases and codon usage table 4 (Mold, Protozoan, and Coelenterate Mitochondrial Code) following Haridas and Gantt (2010), or by comparing with intron-less mtDNA in corresponding insertion sites. The intron types were identified using the RNA-Weasel software (http://megasun.bch.umontreal. ca/RNAweasel) (Lang et al., 2007). The types of intron-encoded ORFs were identified by searching the NCBI conserved domain database. Comparison of introns within same insertion site was performed using the online programs Kalign or Clustal Omega (both can be found at https://www.ebi.ac.uk/Tools/msa/; Li et al., 2015). Insertions of short fragments among these six tested mtDNAs were identified by multiple sequence alignments using Kalign or Clustal Omega. Hairpin structures of short inserted fragments were detected by using the online program Mfold (Zuker et al., 1999). A DNA sequence of hairpin structure with continuous matched stem length >5 and a loop size < 11 was regarded as a small inverted repeat (SIR; Lavrov et al., 2012), and the SIR counts for each genome was tabulated.

## Results

### Species designation

Based on the most parsimonious phylogenetic tree of the concatenation of beta-tubulin and actin gene sequences, 11 strains were clustered into three clades; the *A. stygium* clade, the *A. stygium* var. annulatum clade, and the *A. urceolatum* clade. All six isolates were located in the *A. stygium* clade (Figure S1).

### Assembly of mtDNA

**Figure 1:**
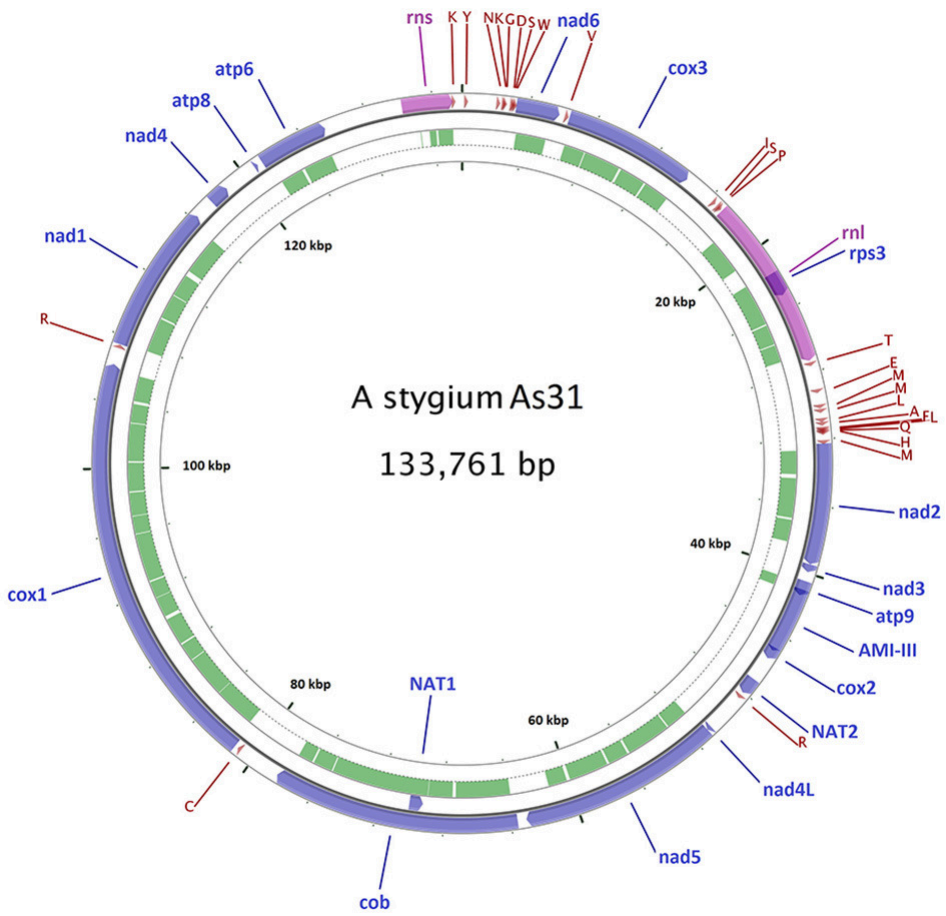
Diagram of the As31 mitochondrial genome. Fifteen conserved protein-coding genes, 26 tRNAs, small and large subunit rRNAs, and three transferase genes are shown. Blue arrows represent protein coding genes; pink arrows represent rRNAs, dark red arrows represent tRNA; green arcs represent introns of conserved protein-coding genes and rRNAs. The other five mtDNAs share similar diagrams as As31, including gene positions, orientation and order.

A 144,034 bp mitochondrial contig was assembled from the 3.5 Gb PacBio reads of As31. Sequence comparison of the ends of the contig showed a 10,285-bp repeat, allowing for joining to a 133,749-bp circular molecule. Twelve singleton indel differences were detected by mapping Illumina reads onto the PacBio assembly. All these differences existed in single nucleotide repeat areas and were corrected to match the Illumina data, finally yielding a mitochondrial genome of As31 with a circular DNA of 133,761 bp (Figure 1).

Using the mtDNA genome of As31 as a reference, mitochondria-related contigs of the five other isolates were extracted from the whole draft genome Illumina assemblies generated using SPAdes-3.8.1. The contigs of As03, As15, As23, As24, and As28 were assembled into circular mtDNAs of 145,449, 131,996, 147,325, 140,328, and 143,806 bp, respectively, leaving one gap in As03 and two gaps in As24 (Figure S2). Primers were designed at the gap ends to bridge the gaps by PCR sequencing. However, the Sanger sequencing did not provide complete sequences, perhaps due to special secondary structures, and the gaps have been filled with N's in the presumed circular sequences.

### Gene content

From the sequence comparisons, the same sets of 15 conserved protein-coding genes, 26 tRNAs, and small and large subunit rRNAs were found in mitochondrial DNAs of all six isolates (Table 1). The conserved protein-coding genes included ATP synthase subunits 6, 8, and 9 (*atp6, atp8*, and *atp9*), the cytochrome oxidase subunits 1, 2, and 3 (*cox1, cox2*, and *cox3*), NADH dehydrogenase subunits 1, 2, 3, 4, 5, 6, and 4L (*nad1, nad2, nad3, nad4, nad5, nad6*, and *nad4L*), apocytochrome b (*cob*), and one ribosomal protein (*rps3* in *rnl*). Twenty-six tRNA genes were comprised of three copies of tRNA-met encoding genes, two for each of tRNA-ser, tRNA-arg, tRNA-leu, and tRNA-lys and the other 15 standard tRNAs, most of which were located between *rnl*-*nad2* (11), *rns*-*nad6* (8), and *cox3*-*rnl* (3). All of these genes were predicted from the same forward strand (Figure 1 for As31). The syntenic order of the conserved genes was identical for all isolates. Most intergenic regions were homogeneous and had similar sizes except for the region between cytochrome b (*cob*) and cytochrome c oxidase I (*cox1*) genes which ranged from 2,998 to 8,039 bp among the six isolates.

**Table 1:** Comparison of mitochondrial genomes of six isolates of *Annulohypoxylon stygium*.

**Isolates**	**Mitochondrial genome size (bp)**	**GC %**	**Conserved protein-coding genes**		**tRNA**		**rRNA**		**intron**		**Intergenic region (bp)**	**Accession**
			**Number**	**Length**	**Number**	**Size (bp)**	**Number**	**Size (bp)**	**Number**	**Size (bp)**		
As03	145,449	30.17	15	15,003	26	1,938	2	4,341	49	87,530	38,059	MH620790
As15	131,996	29.81	15	15,012	26	1,938	2	4,426	47	79,669	32,373	MH620791
As23	147,325	29.77	15	15,015	26	1,938	2	4,431	47	92,443	34,920	MH620792
As24	140,328	30.05	15	15,015	26	1,938	2	4,430	47	85,627	34,740	MH620793
As28	143,806	30.18	15	15,012	26	1,938	2	4,424	48	88,863	34,991	MH620794
As31	133,761	29.86	15	15,012	26	1,938	2	4,427	45	81,437	32,369	NC_023117

*rps3 (1,422 bp) located in intron of rnl, which was counted both as conserved protein-coding gene and intron in the table*.

Three transferase genes as well as a transferase-related fragment were predicted from mtDNA of *A. stygium* As31 (Figure 1). There were two copies of putative N-acetyl-transferase genes located downstream of *cox2* and the third intron of *cob* (*cob*-i3). An aminotransferase class-III like gene (containing two short introns) was in the region between *atp9* and *cox2*, and a fragment encoding AAT_I super family domain was located within the area between *atp6* and *rns*. These genes and fragments were identified in mtDNAs of all six sequenced isolates.

### Intron content and distribution

Different from the conserved genes and transferase genes, the content and distribution of introns within conserved genes varied greatly among isolates (Table 2). Introns within the same insertion site sometimes differed from each other in size and content. Sixty-five intron insertion sites were found in 13 conserved genes: *cox1* was found to have 17 sites; *rnl* had eight; *nad5* had seven each; *cob* and *cox3* had six; *rns* and *nad2* had five each; *nad1* had four; *atp6* had three; and single sites were also found in *nad4, nad6, atp9*, and *cox2*. According to the length and similarity (92% identity), *nad1*-i4 had five different intron types; *cox1*-i2 and *cox1*-i11 contained four types; five introns had three types; 14 introns had two types; and the other 43 introns were singletons. As a result a total of 99 different introns were identified from the six isolates. They varied greatly in size, ranging from 12 bp for *rns*-i2 (As03) to 6,259 bp for *cob*-i3 (As31).

**Table 2:** Information on introns.

**Name**	**Position (aa/bp)**	**Type**	**Presence**						**Length (bp)**	**Lowest similarity**	**Intron type**	**Conserved domain**	**ORF**
			**As03**	**As15**	**As23**	**As24**	**As28**	**As31**					
nad6-i1	80	I	+	+	+	+	+	+	1,911–1,923	97.3	ID	L1	
cox3-i1	73	I	+	+	–	–	+	+	1,330–1,337	94	IB	L1	orf262
cox3-i2	73–76	I	–	–	+	+	–	–	2,496	100	IB	L1; L1	orf262; orf241
cox3-i3	92	I	–	+	–	–	+	+	2,378	100	II	RT	orf547
cox3-i4	143	I	+	+	+	+	+	+	1,681–1,689	99	ID	double L1	orf405
cox3-i5	183	I	+	+	+	+	+	+	1,640–1,649	98.5	IA	double L1	orf362
cox3-i6	214	I	–	–	+	+	–	–	1,779	99.9	IA	L1	orf266
		II	+	–	–	–	–	–	1,743	–	IA	L1	orf254
rnl-i1	305	I	–	–	+	+	–	–	2,562	99.96	II	RVT	orf489
		II	+	–	–	–	–	–	3,048	–	II	RVT	orf625
rnl-i2	510	I	+	–	–	+	–	–	1,530	99.74	I(derived)	GIY-YIG	orf225
rnl-i3	743	I	+	+	–	–	+	–	1,862–1,863	99.7	IC1	GIY-YIG	orf253
		II	–	–	–	+	–	–	521	–	IC1	–	–
rnl-i4	1,086	I	–	–	–	–	+	+	2,289	100	IC1	double L1	–
		II	–	+	+	+	–	–	464–466	99.1	–	–	–
		III	+	–	–	–	–	–	1,022	–	IC1	–	
rnl-i5	1,277	I	+	–	–	–	–	–	1,317	–	IB	L1	orf182
rnl-i6	2,291	I	+	+	+	+	+	+	2,709–2,717	97.7	rps3	–	rps3
rnl-i7	2,372	I	+	–	–	–	–	–	1,640	–	IC2	double L1	orf450
		II	–	+	+	–	+	+	1,314	99.8	IC2	double L1	orf382
		III	–	–	–	+	–	–	1,362	–	IC2	double L1	orf382
rnl-i8	2,428	I	–	+	+	+	+	+	1,144–1,148	99.6	IA	L2	orf261
nad2-i1	125	I	–	+	+	+	+	+	1,487–1,490	98.7	IC2	double L1	orf416
		II	+	–	–	–	–	–	1,454	–	IC2	double L1	–
nad2-i2	210	I	–	+	+	+	+	+	2,548	98.9	II	RT	orf697
nad2-i3	253	I	+	+	+	+	+	+	1,397	99.6	–	double L1	–
nad2-i4	329	I	–	–	–	+	–	–	2,403	–	II	RT	orf711
nad2-i5	566	I	+	–	–	–	–	–	855	–	–	L1	orf207
atp9-i1	60	I	+	–	–	–	–	–	519	–	IA	–	–
		II	–	+	–	–	+	+	667	100	IA	–	–
		III	–	–	+	+	–	–	673–675	99.7	IA	–	–
cox2-i1	77	I	–	+	–	–	+	–	1,134	100	IB	GIY-YIG	orf250
nad5-i1	83	I	+	–	–	–	–	–	2,012	–	–	double L1	orf485
nad5-i2	108	I	+	+	+	+	+	+	1,372	99.4	IC2	double L1	orf376
nad5-i3	138	I	–	+	–	–	–	+	2,895–2,896	99.9	ID	double L1; double L2	–
		II	–	–	+	+	+	–	4,523–4,524	99.9	–	double L1; double L2; GIY–YIG	orf405
nad5-i4	187	I	+	+	+	+	+	+	1,307–1,312	–	IB	L1	orf340
nad5-i5	233	I	+	+	+	+	+	+	2,566–2,574	97.7	ID; ID	–	orf165; orf132
nad5-i6	304	I	+	+	+	+	+	+	1,210	99.6	IB	L1	org380
nad5-i7	468	I	+	–	–	–	–	–	42	–	–	–	–
cob-i1	67	I	–	+	+	–	+	+	3,482–3,489	99.5	IB(5')	L1; L1	orf529; orf461
cob–i2	134	I	+	+	+	+	+	+	1,562–1,564	99.3	ID	GIY-YIG	orf216
cob-i3	143	I	–	–	+	+	–	–	4,153–4,155	99.9	ID; IB	double L1	–
		II	+	+	–	–	+	+	6,251–6,259	99.5	ID; IB	double L1	–
cob-i4	167	I	+	+	+	+	+	+	1,244–1,260	94.7	IA	–	orf222
cob-i5	207	I	+	+	+	+	+	+	933–934	96	ID	L2	orf219
cob-i6	277	I	+	–	–	–	–	–	2,156	–	IB	L2 (b); GIY-YIG	orf296
cox1-i1	59	I	+	–	–	–	+	–	2,858 bp	99.8	II	RVT	orf852
cox1-i2	73	I	+	+	–	–	+	–	1,263	99.4	IB	L1	–
		II	–	–	–	+	–	–	2,561	–	ID; IB	double L1; L1	orf309; –
		III	–	–	–	–	–	+	2,693	–	IC2; IB	double L1; L1	orf401; orf194
cox1-i3	82	IV	–	–	+	–	–	–	4,039	–	ID; IC2; IB	double L1; double L1; L1	orf309; orf417; orf194
		I	+	–	–	–	–	–	2,606–2,822	–	II	RT	orf675
		II	–	+	+	–	+	+	2,676–2,679	100	II	RT	orf675
		III				+			2,822	–	II	RT	orf675
cox1-i4	93	I	+	–	–	–	–	–	2,596	–	IB	double L1	–
cox1-i5	98	I	+	+	+	+	+	+	1,138	100	IB	L1	orf316
cox1-i6	131	I	+	+	+	+	+	+	1,717–1,779	92.9	IB	GIY-YIG	orf275
cox1-i7	149	I	–	–	+	–	–	–	2,682	–	IB	L1; L1	orf326
cox1-i8	174	I	+	–	+	+	–	–	2,465–2,468	96.3	IB	–	orf718
cox1-i9	205	I	–	+	–	–	+	+	1,300	100	ID	double L1	orf173
		II	+	–	+	+	–	–	1,368	100	ID	double L1	orf173
cox1-i10	214	I	+	+	+	+	+	+	1,062	99.3	IB	double L1	orf316
cox1-i11	243	I	+	–	–	–	–	–	4,817	–	IB; ID	L1; double L1	orf404
		II	–	+	–	–	–	–	1,576	–	IB	L1	orf404
		III	–	–	+	–	–	–	3,212	–	IB	L1	orf404
		IV	–	–	–	+	+	+	3,084–3,086	99.2	IB; ID	L1; double L1	orf404
cox1-i12	257	I	+	+	+	+	+	+	1,128	99.3	IB	L1	orf346
cox1-i13	269	I	+	–	–	–	–	–	1,427	–	IB	L1	–
		II	–	+	–	–	+	+	1,495	100	IB	L1	–
cox1-i14	294	I	+	–	–	–	–	–	1,984	100	–	L2	orf501
		II	–	+	+	+	+	+	1,841–1,847	99.5	IB	L1; L2	orf445
cox1-i15	323	I	–	+	+	+	+	+	2,427	98.3	I(derived)	GIY-YIG; GIY-YIG	orf268; orf331
		II	+	–	–	–	–	–	2,474	–	I(derived)	GIY-YIG; GIY-YIG	orf430; orf331
cox1-i16	339	I	+	+	+	+	+	+	1,206–1,229	96.9	I(derived)	double L1	orf277
cox1-i17	397	I	–	+	–	–	+	+	1,607	100	IB	GIY-YIG	orf445
nad1-i1	49	I	–	+	–	–	+	+	2,231	100	IB(3')	GIY-YIG	orf320
nad1-i2	84	I	+	–	–	–	–	–	1,467	–	–	double L1	–
		II	–	+	+	+	+	+	1,594–1,596	97.6	–	double L1	–
nad1-i3	99	I	+	+	+	+	+	+	1,445–1,480	97.6	IC2	double L1	–
nad1-i4	214	I	–	+	–	–	–	+	2,471	100	IB	GIY-YIG (b)	–
		II	–	–	+	–	–	–	3,903	–	IB	GIY-YIG (b); double L1(b)	
		III	–	–	–	+	–	–	3,878	–	IB	GIY-YIG (b); double L1	orf392
		IV	–	–	–	–	+	–	3,843	–	IB	GIY-YIG (b); double L1	orf392
		V	+	–	–	–	–	–	3,896	–	IB	GIY-YIG (b); double L1	orf 327
nad4-i1	152	I	–	–	+	+	–	–	2,138	100	–	double L1	orf302
atp6-i1	120	I	+	+	+	+	+	+	1,490–1,511	96.9	IB	double L1	orf346
atp6-i2	181	I	–	+	–	–	+	+	1,918	100	–	GIY-YIG	orf382
		II	–	–	+	–	–	–	3,269	–	–	GIY-YIG; GIY-YIG (b)	–
atp6-i3	198	I	–	–	+	–	–	–	1,946	–	IC2	GIY-YIG	–
		II	+	–	–	–	–	–	1,905	–	–	GIY-YIG	–
rns-i1	860	I	–	–	+	+	–	–	2,389–2,390	99.9	II	double L1	orf366
rns-i2	910	I	+	–	–	–	–	–	12	–	–	–	–
		II	–	+	+	+	+	+	50	100	–	–	–
rns-i3	1,108	I	–	–	–	+	–	–	1,406	–	IC2	double L1	orf418
rns-i4	1,440	I	–	+	–	–	+	+	432	100	ID	–	–
rns-i5	1,557	I	+	+	–	–	+	+	960–972	97	–	GIY-YIG	–
		II	–	–	+	–	–	–	1,007	–	–	GIY-YIG	–
		III	–	–	–	+	–	–	1,061	–	–	GIY-YIG	–

*Column 2: “position” represents the insertion site of introns into conserved genes; the unit for protein coding genes is aa; the unit for rRNA sequences is bp; interval number means that the insertion site is uncertain, but should be within the interval. Column 3: “type” represents intron type located in same insertion site. Introns with more than 90 % nucleotide identity and similar length are classified in same type. Column 4–9: +/– indicates the presence or not of a corresponding intron. Column 11: “lowest similarity” means the lowest one of similarity values from pairwise intron alignments within same type. Column 13: L1, LAGLIDADG_1 domain; L2, LAGLIDADG_2 domain; RT, reverse transcriptase domain; RVT, RNA-dependent DNA polymerase domain; (b) means degraded domain. Column 14: “ORF” represents intact conserved domain sequences*.

Most introns (86%) belonged to group I or contained group I-characterized conserved domains, which included LAGLIDADG_1, double LAGLIDADG_1, LAGLIDADG_2, GIY-YIG motifs, or mixtures (Table 2). These group I introns were included in all subgroups except IA3. Some introns contained two or more subgroup I specific sequences. For example, subgroup I specific sequences of IB, IC2 and ID were detected in *cox1*-i2 of As23. Among nine group II introns, three carried genes encoding RNA-dependent-DNA polymerase. Interestingly, the group II intron *rns*-i1 only had an orf366 encoding double LAGLIDADG_1 domain. In addition to group I and group II introns, the other five introns were not typical mitochondrial introns. These included *rnl*-i6 (containing the *rps3* gene), *rnl*-i4 (type II) and three short introns (*nad5*-i7 with 42 bp, *rns*-i2 (type I) with 12 bp, and *rns*-i2 (type II) with 50 bp).

The mtDNAs of six isolates shared 19 “common introns,” which appeared in all isolates with similar size and more than 90% identity. Except for *rnl*-i6 (containing conserved gene *rps3*) and *nad5*-i5 (containing two IDs), other “common introns” were group I with the length ranging from 900 to 2 kb. Each contained one conserved domain-encoded ORF or fragment. Different introns from the same insertion sites at 15 positions had different sizes, but shared high sequence similarity (>90%) among partial fragments. Different introns from the same insertion sites of *cox1*-i9, *atp6*-i3, and *atp9*-i1 shared less similarity (80–90% identity) among partial fragments. Two completely different introns were also detected from each insertion site of *cox1*-i14, *rns*-i2, and *rnl*-i1, and *rnl*-i7 (Table 2).

### Differences of short fragments

Forty-four types of short fragments were different among the six tested mtDNAs (Supplemental Table 1). Those fragments ranged from 12 to 106 bp in length, but most (75%) had the size from 30 to 60 bp. Three of them were short introns of *nad5* or *rns*, while 23 fragments were located within introns of the conserved genes. The other 18 fragments were in intergenic regions. Among these short fragments within introns, As23:87773-87824, As24:28235-28282, and As24:37117-37158 were found in LAGLIDADG_1 domain regions; As28:124339-124385, As24:138958-139006 and As24:139007-139060 were in GIY-YIG domain regions. There were three sets of tandem short fragments: As24:138958-139006 and As24:139007-139060; As23:16465-16482 and As23:16483-16545; and As23:118651-118685 and As23:118686-118723.

Out of 44 short fragments, 43 were found in either As03 or one or more of the other five isolates. Seven were found only in mtDNA of As03, whereas 13 co-existed in the other five mtDNAs. Furthermore, three short fragments were found in mtDNAs of As15, As28 and As31, 15 were in As23 and As24, and another five just in As23 or As24. Based on the distribution of short fragments, six mtDNAs were clustered into three groups: group I (AS15, As28 and As31), group II (As23 and As24), and group III (As03). An exception was As28:124339-124385, which was found in mtDNAs of As03, As23, As24, and As28. Twenty seven short fragments had a single SIR (small inverted repeat), three had two SIRs, and one had three SIRs. No typical SIR structures were found in 14 other short fragments.

Some short fragments were multi-copy in individual mtDNAs. They included As03:124881-124916 (8 copies in As03), As23:118651-118685 (7 copies in As23 and As24), As23:118686-118723 (5 copies in As23 and As24), As23:7552-7599 (3 copies in As23 and 2 copies in As24), As24:16469-16531(3 copies in As23 and As24), As24:37117-37158 (3 copies in As23 and As24), As28:35488-35535 (3 copies in As15, As28 and As31). In addition, there were two copies of As23:137527-137604, As23:140496-140543, As23:47023-47067, and As23:80096-80143 in the corresponding mitochondrial genomes. The copy fragments showed more than 90% identity with their corresponding insertions.

### Analysis of twintrons

**Figure 2:**
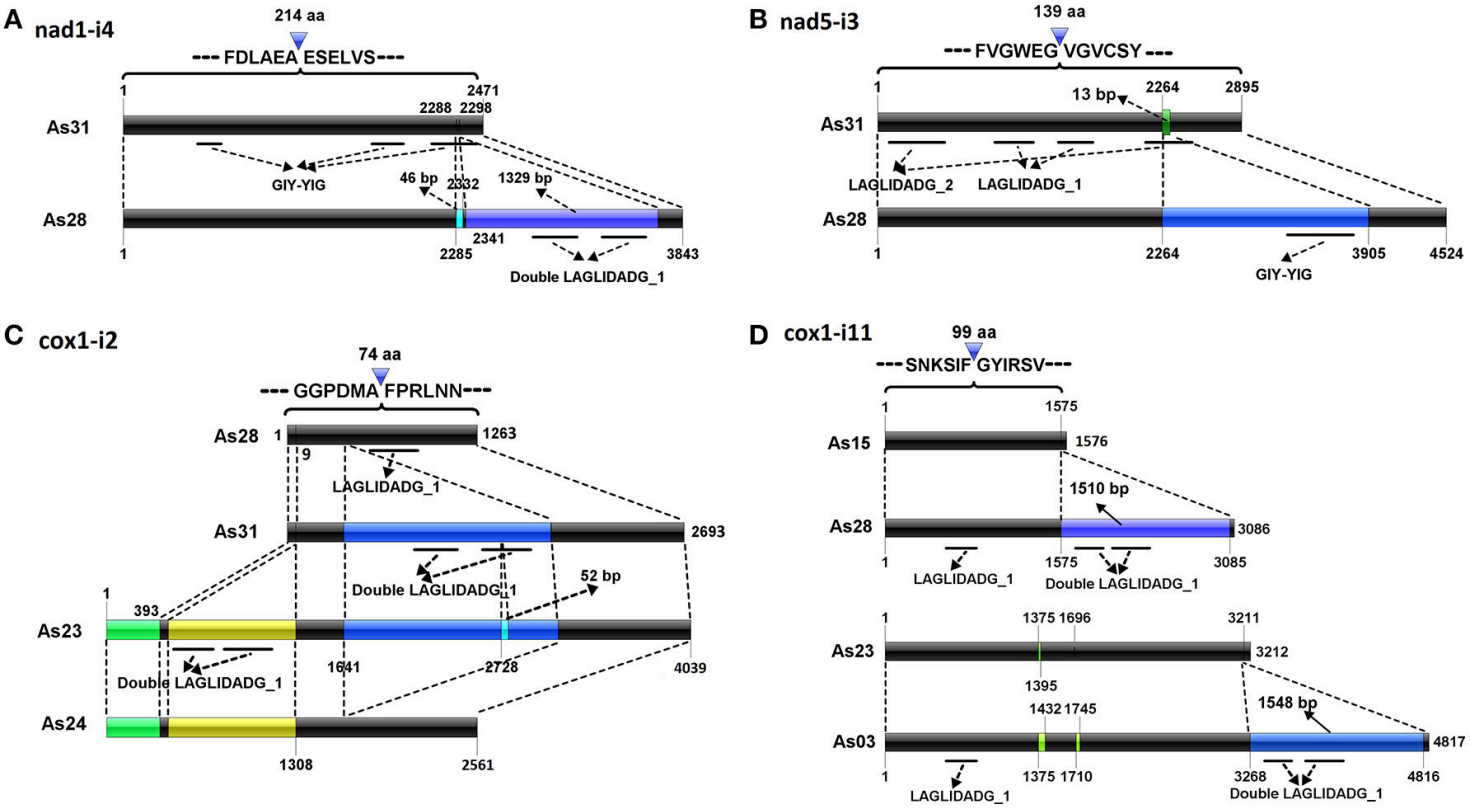
Possible evolution of complex introns. Del operators point to the insertion sites of introns into the conserved genes. Black bars indicate conserved sequences of introns. Other color bars represent new insertion elements. Co-linearity between introns is indicated by dotted lines. Conserved domain regions are shown by solid lines under bars.

Different introns from the same insertion sites shared some parts of similar sequences. Typical cases were located in the sites of *nad1*-i4, *nad5*-i3, *cox1*-i2, and *cox1*-i11. Five different introns found in *nad1*-i4 (Table 1). The *nad1*-i4 of As31 (and As15) was the smallest one (2,471 bp), and contained a GIY-YIG domain encoded sequence, which was interrupted, forming three segments. There were two more fragments by comparing *nad1*-i4 of As28 with that of As31: a 46-bp SIR and a 1329-bp parasitic intron encoding a double LAGLIDADG_1 domain. Parasitic introns are self-splicing introns which insert into another host introns (Gogarten and Hilario, 2006). Both insertions were found in the third GIY-YIG domain segment (Figure 2A). In addition, four small insertions were found in these introns among the isolates: two in As03, one in As23 and As24, and another one in five isolates except As03.

Two introns were detected in *nad5*-i3 (Table 1 and Figure 2B). A smaller one was in each of As15 and As31; a larger one was in each of As23, As24, and As28; and no intron was at that site in As03. The smaller one contained two fragments encoding LAGLIDADG_1 and LAGLIDADG_2 homologs, respectively, indicating that it was a twintron. The larger intron contained an additional 1,500-bp intron, which was GIY-YIG located in a LAGLIDADG_2 coding region. Furthermore, a 13-bp fragment flanked insertion site was found to be lost in larger introns. The larger intron contained the information of at least three introns (Figure 2B).

Four introns varying widely in size were detected in *cox1*-i2, specifically 1,263 bp (As03, As15 and As28), 2,561 bp (As24), 2,693 bp (As31), and 4,039 bp (As23) (Table 1 and Figure 2C). A member of the IC2 intron group with double LAGLIDADG_1 had been inserted into a *cox1*-i2 intron sequence, such as that of As28, to form an intron sequence as found in As31. There were three additional fragments of *cox1*-i2 in As23 compared with that of As31: a 393-bp fragment at its 5′-end, a 909 bp intron with double LAGLIDADG_1 domain, and a SIR in LAGLIDADG_1 domain region. Loss of the fragment from 1,642 to 3,119 bp of a *cox1*-i2 sequence such as in As23, formed a corresponding intron in As24. As a result, three out of four introns in *cox1*-i2 were twintrons found in AS23, As24, and As31, all of which contained the full original intron content of As28.

Four different introns were detected in *cox1*-i11, which were 1,576 bp (As15), 3,086 bp (As24, As28, and As31), 3,212 bp (As23), and 4,817 bp (As03) in length (Figure 2D). They carried a common fragment of approximately 1,300 bp, which contained a LAGLIDADG_1 domain region. Compared with the introns of As15, a parasitic intron with a 1510-bp sequence was inserted into *cox1*-i11 of As28. Similarly, an additional fragment of 1,548-bp was found in *cox1*-i11 of As03 compared to that of As23. The two parasitic introns shared 96.5% similarity.

### Formation of ORF-less intron

**Figure 3:**
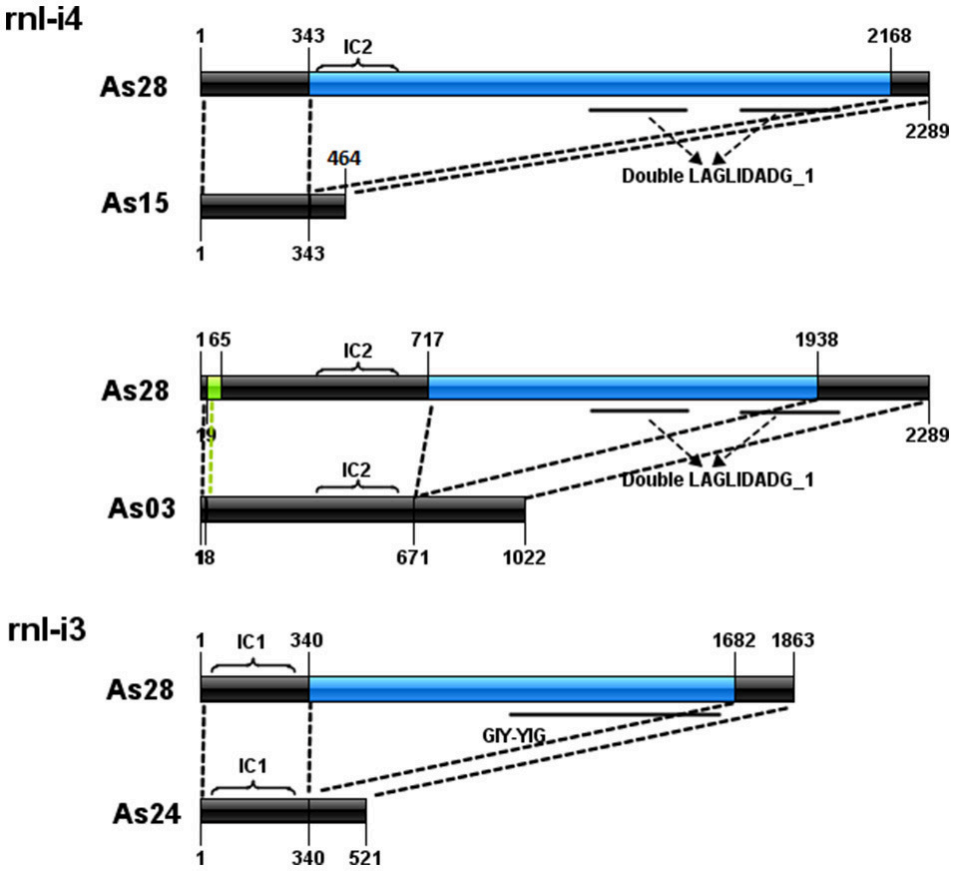
Formation of three orf-less introns. Black bars indicate conserved sequences of introns. Blue bars represent lost parts of sequences. Bright green bars represent short insertion fragments. Conserved domain regions are represented by solid lines under bars.

Comparison of introns at the same loci also showed the formation of three ORF-less introns (Figure 3). The *rnl*-i4 of As28 was a 2,289-bp intron with a subgroup IC2-characterized sequence and a fragment encoding double LAGLIDADG_1 domain. Loss of the fragment from 344 to 2,167 bp to form a 456-bp intron of As15 (without IC2 and double LAGLIDADG_1); loss of the fragment from 717 to 1,938 bp and short fragment from 20 to 64 bp to form a 1,022-bp intron of As03 (without double LAGLIDADG_1). A similar case was found in *rnl*-i3 of As23. A fragment with orf253 encoding a GIY-YIG domain was lost from *rnl*-i3 of As28 to form a 521-bp one of As23.

## Discussion

In this study, we analyzed mitochondrial genomes of six isolates of *A. stygium*. Sixty-five insertion sites and 99 different introns were identified. Introns from each of 16 insertion sites varied greatly in size, but shared high similarity among partial fragments. The similar sequences were inferred to be shared in the common ancestor, and each intron represented one of its evolutionary descendants. Intron comparisons may demonstrate their possible evolution. For *cox1*-i2, as an example, the intron of As28 was a typical mitochondrial intron, with a group IB RNA structure and a LAGLIDAGE_1-like gene. It was inferred to be the ancestor of all introns in *cox1*-i2, since it carried the least content. An additional parasitic IC2 intron was detected in the intron of As31. A 52-bp SIR-like fragment was detected within double LAGLIDADG_1 region of the parasitic IC2 intron of As23, indicating that the intron of As23 may have originated from the ancestral intron currently conserved in As31. Similar to the intron of As23, that of As24 contained two additional fragments at the 5′ end, but lacked the parasitic IC2 intron. The intron of As24 was a presumed descendant of the one from As23, but with the loss of the IC2 intron. As a result, four introns in *cox1*-i2 may have derived from the ancestral homolog from the As28 form, to the As31 form, to the As23 form, and then to the As24 form (Figure 2C).

In a similar fashion, nine twintrons were identified, namely *cox3*-i1 of As24, *nad5*-i3 of As28, *cob*-i3 of As31, *cox1*-i2 of As24, As23, As31, and *cox1*-i11 of As28 and As03 and *nad1*-i4 of As28. Although twintrons are referred to as an intron inserted by one or more parasitic introns (Bullerwell et al., 2003), both insertions of parasitic introns, and activities of short fragments (such as SIRs) or long non-intron fragments can contribute to the formation of twintrons. Activities of non-intronic elements have made the structure of twintrons more complex, which was hard to identify accurately based on the secondary structure of RNA in the host intron. For example, formation of *nad1*-i4 in As28 resulted from at least two insertions: a 46-bp SIR and a 1,329-bp parasitic intron which are found close to each other (9 bp distance; Figure 2A). It was difficult to distinguish the 46-bp SIR and 9-bp host intron fragment from 1,329-bp parasitic intron without comparison with *nad1*-i4 of As31. Mitochondrial introns generally have conserved insertion sites (Swithers et al., 2009). Formation of larger introns in *nad5*-i3 involved an insertion of a 1,500-bp parasitic intron into a smaller intron, as well as the loss of a 13-bp fragment flanking the insertion site. It is unclear if loss of the short fragment was related to the parasitic intron insertion event.

Introns without ORFs have been widely found in fungal mitochondrial genomes (Mullineux et al., 2010; Hafez et al., 2013). HEG is an independent mobile element, which breaks away from intact introns, and results in ORF-less introns (Sethuraman et al., 2013). Intron comparisons in this study revealed two sets of introns with each set containing an intact intron and a corresponding ORF-less one: *rnl*-i3 of As28/As24 and rnl-i4 of As28/As03. Compared with *rnl*-i3 of As28, a 1,341-bp fragment containing a 762-bp GIY-YIG-encoded ORF was lost in As24. The ORF was supposed to be an intact HEG. However, the lost fragment from *rnl*-i4 in As28 just contained part of the double LAGLIDADG_1 motif region, and the other part of which can be found in *rnl*-i4 in As03. Deletion of the fragment was perhaps driven not by mobility of the interrupting HEG, but by other unknown mechanisms.

Several group II introns with ORFs encoding putative LAGLIDADG homing endonucleases have been identified in mitochondrial small or large subunit rRNA genes of both Ascomycetes and Basidiomycetes (Monteiro-Vitorello et al., 2009; Pfeifer et al., 2012). These composite elements were reported to function as group I/HEG composite elements (Mullineux et al., 2010). Here, we also identified a group II (domain V) intron with ORF366 encoding putative double LAGLIDADG HEG in mitochondrial rns gene of As23 and As24.

Aside from introns, the six mtDNA genomes contained the same sets of conserved protein-coding, tRNA and rRNA genes. Arrangement and orientation of these genes were identical. Additionally, intergenic regions except for the region between cob and cox1, were homogenous, and of similar size. Conserved genome profiles, large intron number, and high diversity of intron type, size and number are unique characteristics of mtDNAs of *A. stygium* reported so far, which make them the ideal material for the study of the evolution of fungal mitochondrial introns.

The presence of transferase encoding genes was another feature of mtDNA in *A. stygium*. Transferase encoding genes are rare in fungal mitochondrial genomes, since only three copies were detected from ~500 fungal mtDNAs released in the organelle genome database of NCBI, including an N-acetyl-transferase in *Phialocephala subalpina* (YP_004733051.1), a hypothetical protein of *Pestalotiopsis fici* (YP_009317063.1), and an N-acetyl-transferase of *P. fici* (YP_009317072). However, there were three transferase genes as well as a transferase-related fragment in all six mtDNAs of *A. stygium*. They included two copies of putative N-acetyl-transferase genes and an aminotransferase class-III-like gene. Corresponding transferase genes from different mtDNAs shared high similarity. *P. fici* and *A. stygium* belong to same order of *Xylariales*, and these transferase genes likely evolved via vertical gene transfer from their ancestor, rather than horizontal gene transfer.

The six mtDNAs were 131,996–147,325 bp in length. To our knowledge, only eight completely sequenced and well annotated fungal mtDNAs are larger than 150 kb (Losada et al., 2014; Mardanov et al., 2014; Salavirta et al., 2014; Kanzi et al., 2016; Nowrousian, 2016; Kang et al., 2017). One of the common features for those large mtDNAs was that all of them contain an abundance of introns, many of which carry homing endonuclease genes (group I type) or reverse transcriptase genes (group II type). Those introns occupy a large proportion of their genome, for example up to 67.6% of *O. sinensis* (Kang et al., 2017). Similarly, six mtDNAs in this study contained 45–49 introns, most of which carried self-mobility related genes. Some introns even contained two or more homing endonuclease genes, which made their host intron huge. For example, *cox1*-i2 in *A. stygium* was a large intron of 4,039 bp, which harbored three ORFs with LAGLIDAGE HE genes. Altogether, introns accounted for the range from 59.6 to 62.2% of the full mitochondrial genome sizes among the size isolates. Our findings as well as the previous studies demonstrated that the number and length of introns are two key factors that contribute to the size of large fungal mtDNAs.

Comparative mitochondrial genomic analyses among isolates within intraspecies or closely related species might enhance our understanding of recent fungal mitochondrial evolution. Related researche on several genera has been performed in the past few years. Nine mitochondrial genomes from *Aspergillus* and *Penicillium* species (Joardar et al., 2012), and three from *Cordyceps militaris* (Zhang et al., 2015) represented the small genome sizes (<40 kb); six mtDNAs from *Rhizophagus irregularis* (Formey et al., 2012), five from *Rhynchosporium* species (Torriani et al., 2014), and three from *Fusarium* species (Al-Reedy et al., 2012) represented moderate genome sizes (40 kb to 100 kb); and three out of four mitochondrial genomes from *Chrysoporthe* species (Kanzi et al., 2016) represented large genome sizes (more than 100 kb). Comparisons of isolates from the same genera showed that the mtDNA size of closely related isolates were similar, retaining almost identical synteny of coding gene regions. Most genome size variation can be attributed to the gain/loss of mobile elements such as introns, mobile open reading frames, and mitochondrial plasmid-like DNA polymerase genes. In this study, all six tested mtDNAs were large, and also ranged in size. Similar with previous studies, the main source for size differences of the six mtDNAs was due to mobility of introns plus group II like fragments located at between *cob* and *cox1*.

Common introns were found in all tested mtDNAs, and they could be derived from their ancestors. These introns should be stable in their host genomes and have little or no activity of mobility. Mitochondrial genomes of nine *Aspergillus* and *Penicillium* species have zero common introns (Joardar et al., 2012), of three *Cordyceps militaris* strains have four common introns (Zhang et al., 2015), and of six *Rhizophagus irregularis* isolates have five common introns (Formey et al., 2012). Common intron number (19) of mtDNAs in *A. stygium* was much greater than those species with small mtDNA size, indicating that there might be one or more mechanisms in *A. stygium* which accelerate degradation, and hinder the mobility of their mitochondrial introns.

Small inverted repeats are short elements <100 bp that can fold into hairpin-like structures. These hairpin-like elements have been widely found in the organellar genomes of plants, aquatic animals (Lukić-Bilela et al., 2008; Lavrov, 2010) and fungi (de Zamaroczy and Bernardi, 1986; Dieckmann and Gandy, 1987; Paquin et al., 2000; Formey et al., 2012). Previous studies suggested that small inverted repeats may function in DNA recombination (de Zamaroczy and Bernardi, 1986; Dieckmann and Gandy, 1987), mRNA processing, translation, and stabilization (Yin et al., 1981; André et al., 1992). In this study, 31 out of 44 short insertion fragments contained one or more hairpin structures. There were 18 short insertions among the introns. Six of these were in conserved domain regions in HE genes. The remaining 12 were not found in the conserved domain, but they may still affect the structure of catalytically active introns of RNA, of intron-encoding proteins or of intron insertion sites.

A possible factor for so many introns in mtDNA of *A. stygium* is the presence of short fragment insertions in introns. Insertion of short fragments may result in the change of secondary structure of RNA, altered structure of the conserved domain of HE genes, or interruption of ORF integrity in introns, thus reducing or hindering the activity of RNA or/and HE. Broken introns may increase the frequency of different types of mutations, including insertions/deletions of parasitic introns, loss of HEGs, single base mutation and insertions of other short fragments. All these mutations can give rise to a great number of high-diversity introns, which may contribute to the large genomic size of *A. stygium* mitochondria.

## Conclusions

MtDNAs of six isolates from *A. stygium* ranged from 132 to 147 kb in size and contained the same sets of conserved protein-coding, tRNA and rRNA genes, and shared the same gene arrangement and orientation. Those six genomes varied greatly in intron distribution and content. Comparison of introns within the same insertion site demonstrated formation of some complex introns, such as twintrons and ORF-less introns. Complex introns underwent not only insertion/deletion of introns, but also insertion/deletion of short fragments such as small inverted repeats. Movements of short fragment within an intron might affect or hinder the activity of introns, which could cause intron accumulation in mitochondrial genomes, and enlarge their size.

## Author contributions

BX and RM conceived this study. YD analyzed the data, prepared figures and drafted the manuscript. TH participated in early analysis of preliminary data and manuscript writing and revision. SL provided suggestion for the research, contributed to the data interpretation, writing, and revising the manuscript critically. QC participated in the data analysis. LL and QW sequenced and analyzed the mitochondrial genome. All authors have read and approved the final version of the manuscript.

### Conflict of interest statement

The authors declare that the research was conducted in the absence of any commercial or financial relationships that could be construed as a potential conflict of interest.

## Supplementary material

Figure S1 Phylogenetic tree of six isolates of *Annulohypoxylon stygium* based on partial beta-tubulin and actin gene sequences. Concatenation of beta-tubulin and actin gene sequences was used for species designations of the isolates. Corresponding sequences of *Annulohypoxylon urceolatum* (AY951670 and AY951782), *A. stygium* 90081906 (AY951667 and AY951775), *A. stygium* 90041409 (AY951666 and AY951776), *A. stygium* var. annulatum 3 (AY951669 and AY951777) and *A. stygium* var. annulatum 91042205 (AY951668 and AY951778) were used. Sequences were aligned using Clustal W with gap opening penalty of 5 and gap extension penalty of 2. The distances were computed using the Neighbor-joining algorithm as implemented in Mega 7, and all positions containing gaps and missing data were eliminated from phylogenetic analysis.

Figure S2 Diagram of six mitochondrial genomes. Fifteen conserved protein-coding genes, 26 tRNAs, small and large subunit rRNAs, and three transferase genes are shown. Blue arrows represent protein coding genes; pink arrows represent rRNAs, dark red arrows represent tRNA; green arcs represent introns of conserved protein-coding genes and rRNAs.
