# Supplementary material for: Comparison of the Mitochondrial Genome Sequences of Six Annulohypoxylon stygium Isolates Suggests Short Fragment Insertions as a Potential Factor Leading to Larger Genomic Size
Source: Front Microbiol. 2018 Sep 10;9:2079. doi: 10.3389/fmicb.2018.02079 (PMC6140425; doi:10.3389/fmicb.2018.02079)
Supplement: Supplementary file 1 [file Table_1.docx]

**Supplemental Tables**

**Table S1 |** Information on short fragments. +/- indicates the presence or not of a corresponding short fragment.

| **No.** | **Type** | **Length** | **Location** | **As03** | **As15** | **As23** | **As24** | **As28** | **As31** | **SIR structures** |
| --- | --- | --- | --- | --- | --- | --- | --- | --- | --- | --- |
|  |  |  |  |  |  |  |  |  |  |  |
| 1 | As23:7552-7599 | 48 | Cox3-i1 | - | - | + | + | - | - | 1 |
| 2 | As23:13897-13932 | 36 | Cox3-i6 | - | - | + | + | - | - | 1 |
| 3 | As24:16469-16531 | 63 | Cox3-rnl | - | - | + | + | - | - | 0 |
| 4 | As23:16465-16482 | 18 | Cox3-rnl | - | - | + | - | - | - | 0 |
| 5 | As15:19967-20013 | 47 | rnl-i4 | - | + | + | + | + | + | 1 |
| 6 | As24:28235-28282 | 48 | rnl-i7 | - | - | - | + | - | - | 1 |
| 7 | As15:27625-27660 | 36 | rnl-nad2 | - | + | + | + | + | + | 1 |
| 8 | As15:28027-28066 | 40 | rnl-nad2 | - | + | + | + | + | + | 1 |
| 9 | As15:29987-30058 | 72 | rnl-nad2 | - | + | + | + | + | + | 2 |
| 10 | As23:33465-33504 | 40 | rnl-nad2 | - | - | + | + | - | - | 1 |
| 11 | As28:35488-35535 | 48 | nad2-i1 | - | + | - | - | + | + | 1 |
| 12 | As24:37117-37158 | 42 | nad2-i1 | - | - | + | + | - | - | 1 |
| 13 | As24:104132-104173 | 42 | cox1-i14 | - | - | + | + | - | - | 1 |
| 14 | As23:47023-47067 | 45 | cox2-nad5 | - | - | + | + | - | - | 1 |
| 15 | As23:47816-47873 | 58 | cox2-nad5 | - | - | + | + | - | - | 0 |
| 16 | As23:49420-49525 | 106 | cox2-nad5 | - | - | + | + | - | - | 0 |
| 17 | As23:66104-66145 | 42 | cob-cox1 | - | - | + | + | - | - | 0 |
| 18 | As15:79641-79690 | 50 | cob-cox1 | - | + | + | + | + | + | 1 |
| 19 | As15:81157-81189 | 33 | cob-cox1 | - | + | + | + | + | + | 1 |
| 20 | As15:82069-82103 | 41 | cob-cox1 | - | + | - | - | + | + | 0 |
| 21 | As03:84000-84092 | 93 | cob-cox1 | + | - | - | - | - | - | 3 |
| 22 | As23:80096-80143 | 48 | cob-cox1 | - | - | + | + | - | - | 1 |
| 23 | As23:81928-81995 | 68 | cob-cox1 | - | - | + | + | - | - | 1 |
| 24 | As23:87773-87824 | 52 | cox1-i2 | - | - | + | - | - | - | 1 |
| 25 | As15:86349-86420 | 72 | cox1-i3 | - | + | + | + | + | + | 0 |
| 26 | As03:106290-106325 | 36 | cox1-i11 | + | - | - | - | - | - | 1 |
| 27 | As23:104231-104256 | 26 | cox1-i11 | - | - | + | - | - | - | 0 |
| 28 | As03:108248-108289 | 42 | cox1-i11 | + | - | - | - | - | - | 2 |
| 29 | AS15:98988-99039 | 52 | cox1-i15 | - | + | + | + | + | + | 0 |
| 30 | As15:108571-108654 | 83 | nad1-i2 | - | + | + | + | + | + | 1 |
| 31 | As15:108843-108890 | 48 | nad1-i2 | - | + | + | + | + | + | 0 |
| 32 | As23:118651-118685 | 34 | nad1-i4 | - | + | + | + | + | + | 1 |
| 33 | As23:118686-118723 | 39 | nad1-i4 | - | - | + | + | - | - | 1 |
| 34 | As28:124339-124385 | 46 | nad1-i4 | + | - | + | + | + | - | 1 |
| 35 | As03:124881-124916 | 36 | nad1-i4 | + | - | - | - | - | - | 1 |
| 36 | As03:125054-125111 | 58 | nad1-i4 | + | - | - | - | - | - | 2 |
| 37 | As15:121712-121745 | 34 | atp6-i2 | - | + | - | - | + | + | 1 |
| 38 | As23:137527-137604 | 78 | atp6-rns | - | - | + | + | - | - | 0 |
| 39 | As24:138958-139006 | 49 | rns-i5 | - | - | + | + | - | - | 1 |
| 40 | As24:139007-139060 | 54 | rns-i5 | - | - | - | + | - | - | 1 |
| 41 | As23:140496-140543 | 48 | rns-end | - | + | + | + | + | + | 1 |
| 42 | As03:63680-63722 | 42 | is nad5-i7 | + | - | - | - | - | - | 0 |
| 43 | As15:129332-129381 | 60 | is rns-i2 | - | + | + | + | + | + | 1 |
| 44 | As03:143141-143152 | 12 | is rns-i2 | + | - | - | - | - | - | 0 |
