# Supplementary figures and images for: Comparison of the Mitochondrial Genome Sequences of Six Annulohypoxylon stygium Isolates Suggests Short Fragment Insertions as a Potential Factor Leading to Larger Genomic Size
Source: Front Microbiol. 2018 Sep 10;9:2079. doi: 10.3389/fmicb.2018.02079 (PMC6140425; doi:10.3389/fmicb.2018.02079)

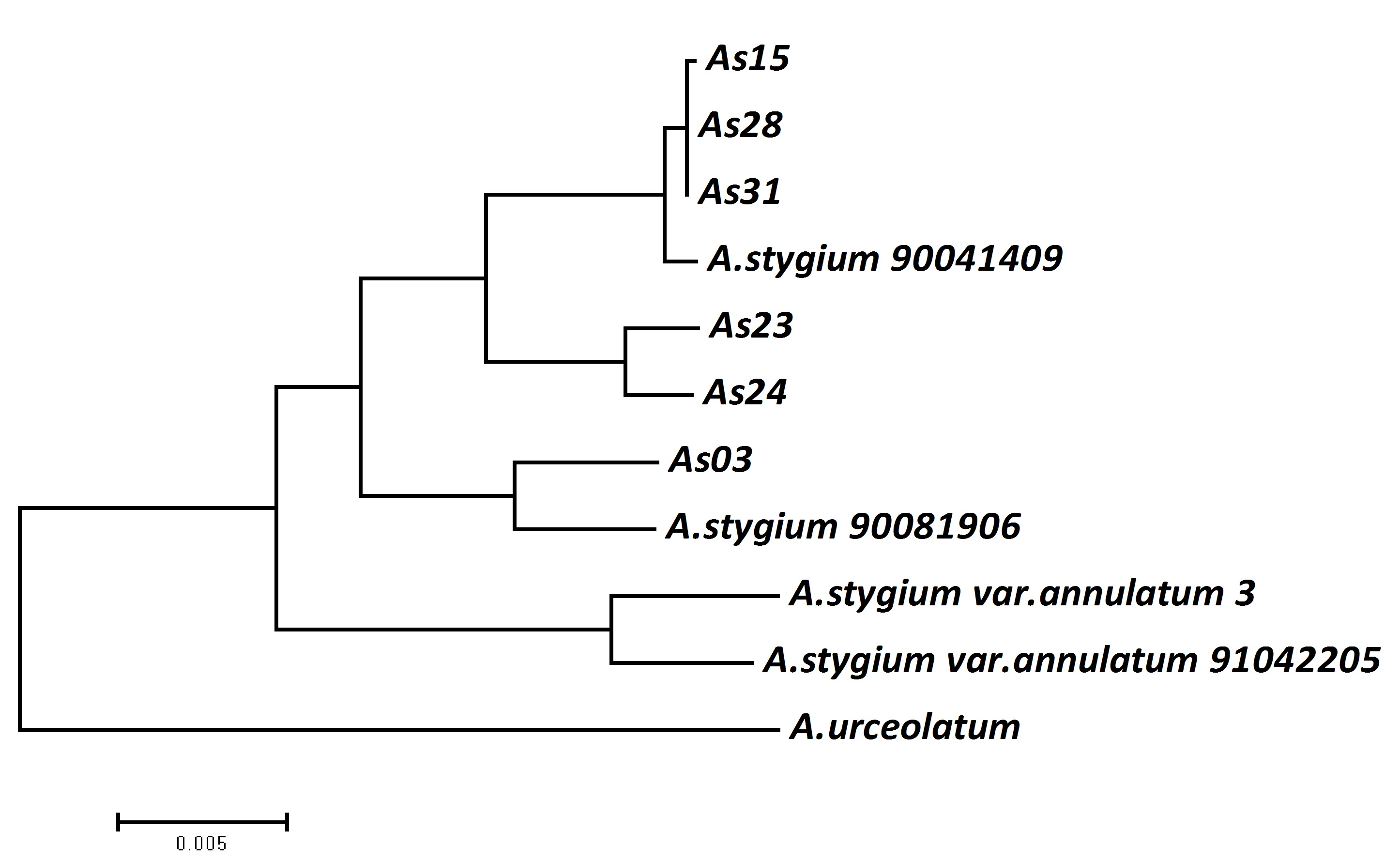

Supplement: Figure S1 — Phylogenetic tree of six isolates of Annulohypoxylon stygium based on partial beta-tubulin and actin gene sequences. Concatenation of beta-tubulin and actin gene sequences was used for species designations of the isolates. Corresponding sequences of Annulohypoxylon urceolatum (AY951670 and AY951782), A. stygium 90081906 (AY951667 and AY951775), A. stygium 90041409 (AY951666 and AY951776), A. stygium var. annulatum 3 (AY951669 and AY951777) and A. stygium var. annulatum 91042205 (AY951668 and AY951778) were used. Sequences were aligned using Clustal W with gap opening penalty of 5 and gap extension penalty of 2. The distances were computed using the Neighbor-joining algorithm as implemented in Mega 7, and all positions containing gaps and missing data were eliminated from phylogenetic analysis. [file Image_1.JPEG]

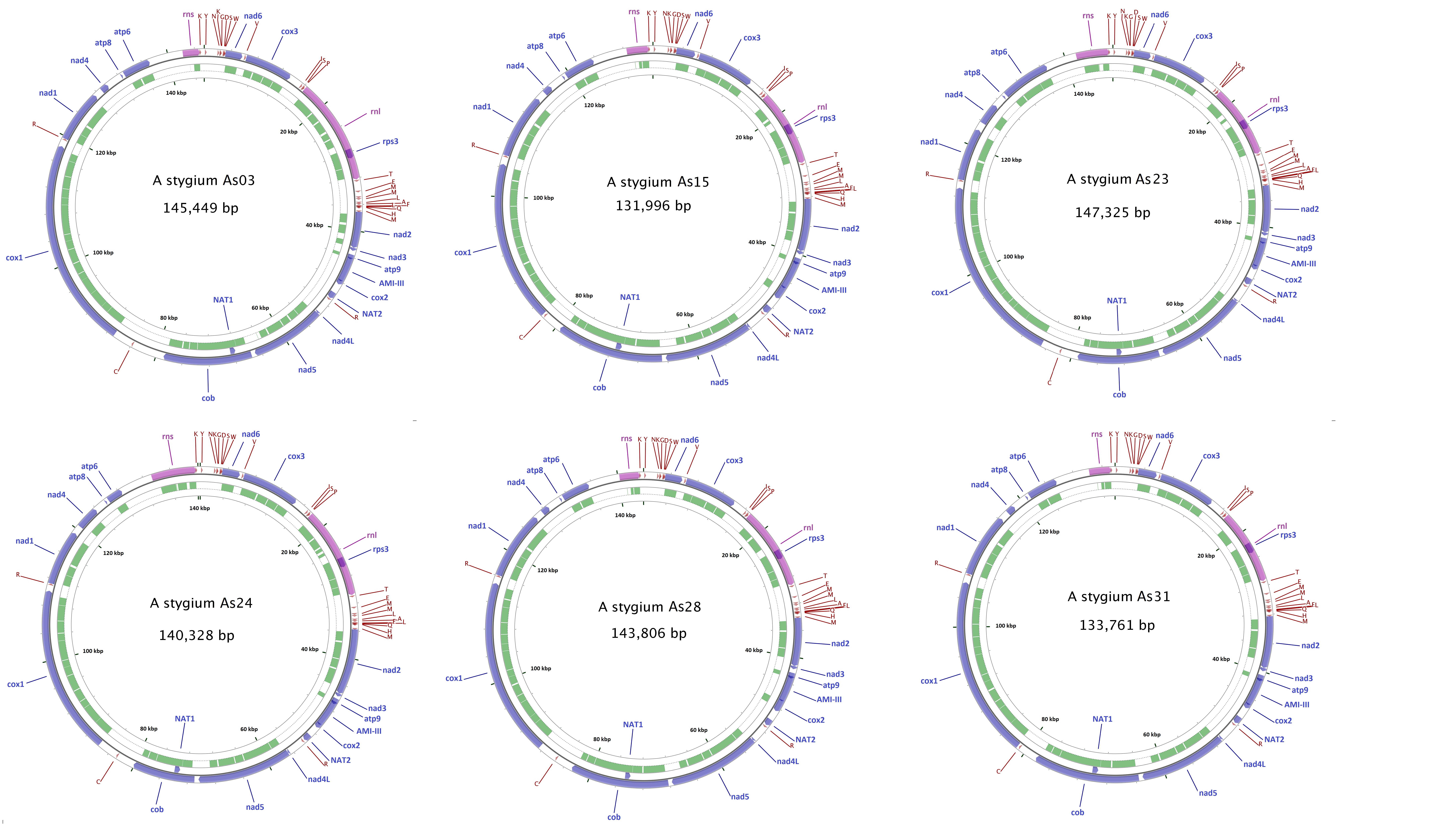

Supplement: Figure S2 — Diagram of six mitochondrial genomes. Fifteen conserved protein-coding genes, 26 tRNAs, small and large subunit rRNAs, and three transferase genes are shown. Blue arrows represent protein coding genes; pink arrows represent rRNAs, dark red arrows represent tRNA; green arcs represent introns of conserved protein-coding genes and rRNAs. [file Image_2.JPEG]
